# Wraparound Supports: The Key to the Transition and Integration of Internationally Educated Registered Nurses in Rural Alberta, Canada

**DOI:** 10.1155/jonm/7378466

**Published:** 2026-04-21

**Authors:** Monique Sedgwick, Helen Kelley

**Affiliations:** ^1^ Faculty of Health Sciences, University of Lethbridge, Lethbridge, Alberta, Canada, uleth.ca; ^2^ University of Lethbridge, Lethbridge, Alberta, Canada, uleth.ca

**Keywords:** acculturation, host countries, integration, internationally educated nurses, overseas trained nurses, rural communities, rural nursing, transition, wraparound support

## Abstract

**Background:**

Internationally educated nurses (IENs) face multiple challenges during their transition and integration to nursing settings in host countries. Two foci are prevalent in the extant literature: IENs’ experiences in urban nursing settings and internationally educated healthcare professionals’ experiences. There is a distinct gap with understanding IENs’ experiences while transiting and integrating into rural nursing practice and communities.

**Objective:**

This study aimed to understand IENs’ experience of rural nursing practice and the strategies they used to enhance their transition and integration into the rural workforce and community settings.

**Method:**

Semistructured interviews and participant photographs representing their experiences from the qualitative phase of a sequential mixed methods design were conducted in Alberta, Canada.

**Findings:**

The transition and integration of IENs depend upon the wraparound support they received through interactions and connections with healthcare leaders, managers, administrative members, nursing colleagues, and community members. Embedded in these interactions and connections were various strategies IENs used to help them navigate and manage their desire to “fit in” and to be accepted in their rural nursing and community settings.

**Conclusion:**

IENs were highly motivated to become accepted as contributing members of the nursing team and rural communities. To achieve this goal, they engaged in behaviors to become a valuable member of the nursing team by acquiring additional practice knowledge and skills to enhance their competencies. They also used strategies to connect and “fit into” their new community. Wraparound supports included not only the employer and nursing colleagues but also key community members with social capital. These supports were key to IENs’ engagement in and navigation of the transition and integration processes.

**Implications for Nursing Management:**

Healthcare leaders, managers, and key community members play pivotal roles in the development of cross‐cultural, inclusive work, and living places by providing wraparound supports that reduce the cultural and rural practice distance between IENs and their nursing colleagues as well as creating opportunities for establishing cultural‐sensitive work and living environments.

## 1. Introduction

Canada continues to experience a critical shortage of nurses with job vacancies (registered nurses (RNs), psychiatric nurses, and licensed practical nurses (LPNs)) ranging from 30,970 in the first quarter to 26,430 in the third quarter of 2025 [1]. The long‐term picture of the nursing shortage reinforces discussions about the unsustainability of healthcare delivery in Canada given that the predicted shortage of nurses in Canada is estimated to be 117,600, or 26.3% of supply, by 2030 [2]. In 2022, internationally educated nurses (IENs) made up approximately 12% of the Canadian nursing workforce [3]. In 2024, the Canadian Institute for Health Information (CIHI) reported an increase to 13.3% of IENs (RNs) in the nursing workforce [4]. However, a recent government report highlights that IENs are “underutilized workers” in healthcare because many are employed outside of healthcare or in healthcare occupations with lower education requirements than the IENs had obtained in their home country [5]. To address the threat to the sustainability of the rural nursing workforce where the shortage is more evident [6], Alberta Health Services^1^ (AHS) and the College of Registered Nurses of Alberta^2^ joined forces to reduce barriers to entering the provincial nursing workforce by streamlining the registration process and enhancing onboarding of new hires.

A cursory review of the IEN literature indicates that their motivation behind immigration is multifaceted and may include perceived financial gain [7] through better career opportunities and potential advancement [8], and escape from political instability, violence, and crime in their home country [9]. There is also evidence that IENs experience challenges in the host country following immigration. These challenges include the lack of professional development opportunities [10]; feeling that their nursing knowledge, skill, and competence are undervalued or devalued [11]; being hired in the LPN role in care facilities or long‐term care homes where they cannot fully use their knowledge and skills in their practice due to limited tasks and responsibilities [12]; experiencing discrimination and marginalization because of communication barriers [13]; and difficulty adjusting to the legislated scope of practice [14]. Additionally, since IENs may come from countries where the healthcare system is different than the host country [15], they may experience confusion and misunderstandings not only with host nursing and medical staffs but also with the public because of linguistic racism [16, 17].

A review of the rural nursing literature reveals a gap pertaining to IENs’ experiences of rural nursing practice. While RNs who work in urban hospitals may possess advanced nursing knowledge and skill for the provision of nursing care to specific and unique populations like pediatric patients, high‐risk labor and delivery patients, or surgical patients, rural nurses tend to have a broader practice lens. Nurses who work in rural settings are frequently described as “expert generalists” providing care to patients across the lifespan and health challenges [18–20]. Changes to legislation, professional regulation, and education requirements [21] have resulted in an expansion in their scope of practice to meet the needs of the patient population [22]. Moreover, the broader practice lens is highlighted even further since nursing practice becomes more generalized the smaller the rural hospital, suggesting that there is constant evolution of rural nurses’ scope of practice [20, 22, 23].

From a psychosocial perspective, IENs living and working in rural settings where diversity in language and communication styles, religious ceremonies, and ethnocultural celebrations are confined to the private sphere, learning how to cope with and navigate a monocultural ethos [24] may be very difficult. Indeed, since community members and healthcare teams tend to be “close knit” and share deep personal and professional ties, managers, nursing staff and members of the interdisciplinary team may be unaware of the challenges IENs navigate [25] to become a member of the healthcare team and rural community. Consequently, loneliness and professional and personal isolation [26] may be exacerbated for rural IENs.

To help situate the study, it is important to define “rural”. The exact criteria for “rural” are a matter of some contention since there currently is no consensus on a singular definition of “rural” [27]. However, the concept often includes a combination of geography, culture, population size or density, and qualitative experience [28]. Herron and Skinner recommend a relational approach to studying rural health because it acknowledges that “people, ideas, and goods flow between urban and rural settings, connecting people and places in diverse ways and across a range of scales” [[29], p. 268]. For this study, we understood rural as being relational. The aim of this paper, therefore, is to present the experiences of newly recruited IENs living and working in rural Alberta, and the strategies they use to “fit in” to become valuable contributors to nursing practice and their rural community through key interactions.

## 2. Materials and Methods

### 2.1. Study Design and Context

This study used a sequential mixed methods approach consisting of individual qualitative interviews and a cross‐sectional online survey. To set the context for the qualitative interview, participants were asked to take photographs that represented their experience of living and working in a rural setting. Photographs included events, people (excluding patients and patient information), spaces, and objects and highlighted participants’ reality and their expressions of their lived experiences. Along with the interview guide where questions evolved interviews progressed, the photographs and accompanying written narratives were used as a mechanism to stimulate each participant’s reflection of their experience of rural nursing practice and rural living as well as their interpretation of the underlying meanings of their experiences.

### 2.2. Ethics

The study was approved by the University of Alberta Research Ethics Office (Pro00137602) and received operational approval from AHS. All members of the research team signed a confidentiality agreement. A professional transcription company was hired to transcribe the interviews which were uploaded to their secure site. Transcriptionists followed company privacy and confidentiality policies, which aligned with the ethics protocol. Written consent, via DocuSign, was obtained from each IEN who participated in the qualitative interviews. They were asked to select a pseudonym to protect their privacy and anonymity. Participant quotes are followed by the participant’s pseudonym. Written consent was granted by the participants as per the ethics protocol and AHS operational approval guidelines to use photographs taken by them for scholarly dissemination activities. AHS operational approval restricted photographing patients and related patient information. Supporting file 1 provides the written consent form that outlines the use of photographs. Participants’ characteristics are reported at the aggregate level to further protect participant privacy and anonymity of their responses.

### 2.3. Participants

Our sample consisted of a subset of newly recruited international nurses, screened and assessed by CRNA to ensure that they had the skills and experience to work as a RN in Alberta. They arrived in groups over several months in 2023 and 2024. Because of AHS operational approval restrictions, the eligible pool of potential participants in rural facilities was limited to 23 IENs. These IENs received the recruitment email via the Chief Nursing Office in AHS. Although initial interest in the study generated several inquiries, three participants who initially agreed to participate in the interview withdrew from the study stating they had concerns with taking photographs. Two other IENs expressed interest in participating in the interview, but they did not respond to invitations for the individualized training session pertaining to taking photographs. Seven IENs, approximately 30.4% of the eligible pool, participated in the qualitative component of this study. Table 1 presents the sampling details.

**TABLE 1:** Sampling details.

Sampling	Description
Sampling frame	^∗^Newly recruited IENs who were hired to work as a RN in a rural healthcare facility in Alberta.

Sampling strategy and inclusion criteria	^∗^Purposeful sampling included characteristics aligned with the sampling frame, including the inclusion criteria: • IENs with similar work environments; • Recruited to rural nursing practices in 2023 and 2024; • Similar duration in rural nursing practice; • Participated in similar recruitment and orientation procedures; and • Working in an operationally approved site.

Exclusion criteria	^∗^Included the following: • Working in rural nursing practice at nonapproved sites; • Working in urban nursing practice; • Hired by AHS before 2023; and • Not employed by AHS.

Recruitment procedures	^∗^Required to follow AHS’ approved procedures: • IEN sample generated by AHS, and • Chief Nursing Office of AHS distributed recruitment and reminder emails between September 2023 to September 2024.

### 2.4. Procedures and Data Collection

Once the consent form was signed and returned to the researchers via DocuSign, interviews were scheduled. Audiorecorded semistructured, individual interviews were conducted via Zoom. The interview guide including probing questions, and the supplementary participant photographs and narratives that were arranged in a PowerPoint presentation, provided the structure of the interview. Demographic questions were asked at the end of the interview with data being recorded in a Word document. When available, a graduate student took notes during the interview. Each interview lasted approximately 60 min. Once the interview was completed, it was uploaded to a secure environment where only members of the research team had access. Recordings were then uploaded to the professional transcription company site and transcribed by a professional transcriptionist. Once returned to the principal researcher, the transcripts were reviewed by two graduate students for accuracy and completeness. Consistent with a qualitative approach, initial questions used in the interview arose from our understanding of the literature and personal experiences of living and working in rural settings. As the interviews progressed, some questions were modified a bit, some questions were not asked, and new questions were asked. The interview guide that guided initial interviews is outlined in Table 2. During the interviews, the photographs and related narratives were used to facilitate the discussion and as a catalyst and probe to generate deeper conversations about participants’ experiences.

**TABLE 2:** Interview guide.

*Questions Regarding Transition to Living in Rural Community*
Describe for me what your experience moving to a small rural community has been like for you.
Describe for me what your family’s (spouse, children, parents) experience moving to a small rural community has been like for them.
• How were they impacted?
• How were they impacted by your employment?
• Has your relationship with your family changed since the move and if so, how?
• Have their expectations been met? How so?
• Have your expectations been met? How so?

*Questions Regarding Nursing Practice*
Describe for me what your experience working in a rural hospital has been like.
• Describe for me what has been the most challenging aspect of your scope of practice.
• Describe for me what has been the least challenging aspect of your nursing practice.

*Questions Regarding Transition to Rural Nursing Practice*
• What steps/actions/behaviors did you take to enhance your resilience (ability to cope/adjust/adapt) while working in a rural nursing practice?

*Questions Regarding Integration into the Rural Community*
• What steps/actions/behaviors did you take to facilitate your integration and acceptance into the community?

*Questions Regarding How Their Experience Could be Enhanced*
• What recommendations would you make to enhance/improve your experience of moving to Alberta to work as a rural nurse?

*Questions About Photographs and Narratives*
• What is the photograph about?
• How does this photograph represent your experience of working/living in rural Alberta?
• Why is this photograph important to you?

*Is there anything else you would like to share with me about your experience of being a nurse in a rural hospital?*

### 2.5. Data Analysis

The researchers independently conducted inductive thematic analysis of the qualitative data. They independently immersed themselves in the data by reading and rereading the transcripts and field notes, and by reviewing the photographs and associated narratives. A standardized multistep protocol was followed to identify patterns and themes emerging from the qualitative data (Table 3).

**TABLE 3:** Data analysis protocol.

Procedures	Steps
Independent analysis	1. Read and reread transcripts for overall understanding and review the photographs and related narratives. 2. Generated initial codes. 3. Grouped similar codes into categories and subcategories. 4. Refined categories and subcategories. 5. Identified and defined tentative themes supported by categories and subcategories. 6. Analyzed themes in relation to study’s research questions.

Team review and discussion (3 cycles)	7. Reviewed and discussed their independent findings. 8. Reached consensus on emergent themes, categories and subcategories. 9. Agreed on themes, categories and subcategories: extremely high level of agreement between two researchers. 10. Repeated steps 8 and 9.

Last procedure	11. Map themes and their definitions, and categories and subcategories with relevant quotes to summary table.

## 3. Results

### 3.1. Demographic Results

IEN participants immigrated from Asia, Africa, and North America. They ranged in age between 25 and 44 years. All were married. Three participants identified as female, and four as male. For most of the participants, their spouse and children immigrated with them. All the participants reported that the language of instruction for their nursing program was English. Participants’ years of nursing practice experience ranged from 6 to 20 years prior to immigrating to rural Alberta. Only two participants had prior experience working in a rural setting in their home country. Study participants were relatively new to rural Alberta and reported their employment duration from 3 to 9 months.

### 3.2. Qualitative Results

Thematic analysis identified that IENs depend on key interactions when navigating their transition and integration into rural nursing practice and workplace, and the rural community. Meaning is given to these interactions through the supports they receive from community members and healthcare team. Direct quotes from the participants are provided to bring their experiences to the foreground. Photographs have been inserted to add additional context about IENs’ experiences. These crucial interactions and supportive behaviors appear to be a new finding in the literature, which thus far has focused, almost exclusively, on IENs working in urban settings.

#### 3.2.1. Workplace Community Interactions

**FIGURE 1:**
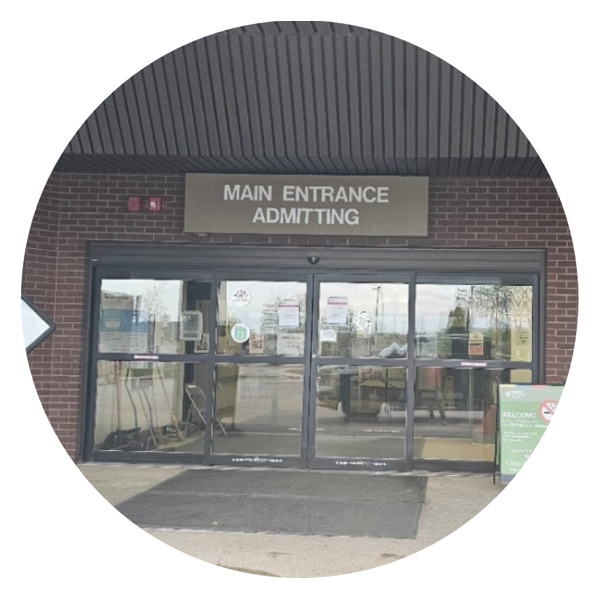
Participant’s title: the first day. Photograph used with written consent.

IENs are highly committed to making their transition and integration to rural nursing practice successful. From their initial interactions and during their first few months in the rural workplace setting, managers and, indeed, the whole healthcare team including members of the organizational leadership team had the power to set the tone of what the participants’ experiences would be like (Figure 1).

While most participants had positive experiences with managers and the organization’s recruitment and leadership teams, they also indicated that at times information was lacking. Unfortunately, interactions that did not address their needs or lacked transparency had the potential to leave participants feeling adrift, confused, and disillusioned.

Initially, when I started, I really loved what I was doing. But the sad part about it is, I worked for five months, and I was never independent. They just kept me on orientation shifts, not really doing anything, not really guiding me but they just never approved me. I am able to work independently in papers and initially I was working independently. I had almost seven years of experience back in [my home country], so I was kind of familiar with all the procedures, and I did know how to access resources. Despite that, I never got the liberty to work on my own, I was being orientated all the time. It was very devastating. I did not feel good at all, I just wanted to leave because I was not feeling that was the way I should be treated. I feel like that was not fair for me, because if I am making mistakes, if I am doing something wrong then I will take that on me. But if I am not making mistakes [and], if in your documentation, you are saying that “you are doing great” then why is that thing happening to me? And I was not happy at all: for mental health, I was not feeling very good about it. I feel like my mental health was kind of compromising because of that (Lisa).

**FIGURE 2:**
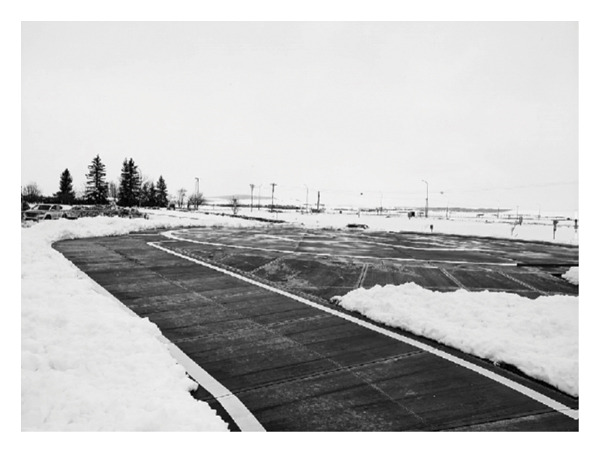
Participant’s title: “a lifeline.” Photograph used with written consent.

**FIGURE 3:**
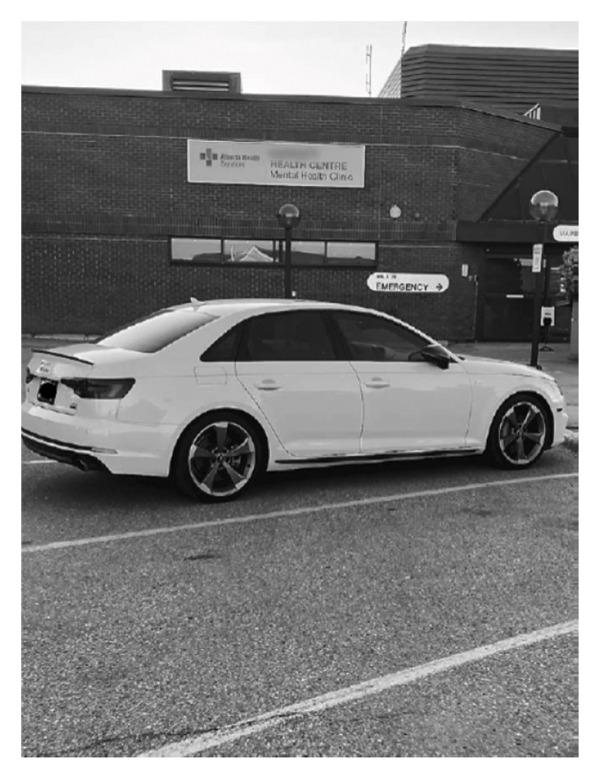
Participant’s title: “coming together to avoid a tragedy.” Photograph used with consent.

Consistent with the literature, study participants also talked about how their scope of practice changed to meet the healthcare needs of community members. For the participants, this meant working *“in all spaces”* (possible), that is, in multiple patient care areas with patients across the lifespan, who have a myriad of health challenges. They also quickly realized that there were limited resources (human and material) which meant they identified “*lifelines”* (Jason) like telephones, text messaging, and access to paramedics and a helicopter landing pad (see image below). Moreover, participants quickly learned that interactions with members of the interdisciplinary team, including police members and community members, were essential for the provision of safe patient care (Figures 2 and 3).

Scope of practice, how do I say? Here we do lots, we do tons of assessment, we do tons of figuring things out. Before you even call the doctor, you have to do your full assessment first before even bothering the doctor. Back [home], you can’t do anything without doctor’s orders. Coming in as a fresh rural nurse, there’s this thing in me that, “Do I need to call the doctor now, or do I need to do this first?” So, before I even call the doctor, I have to be really sure I’ve done everything in my scope, I’ve done all the assessments so that I could to say “OK, this patient needs a doctor right away” (Jason).

Why take a picture of a car in a hospital parking lot, one might ask. Well, an unforgettable incident of a 3‐month‐old baby being accidently locked in the car by his young visibly exhausted mother occurred in that lot. What warmed my heart is how the community came together to quickly solve this issue. A nurse, an RCMP officer, a locksmith and a member of the community worked together and got the door open at no cost or damage to the vehicle. Baby was all smiles as was his mother! (Diana).

Along with a wider scope of practice, lack of familiarity with patient care equipment and differences in responsibilities required the participants to adjust their practice and develop an understanding of cultural differences within their work environment so that they could be successful in integrating into the healthcare team. These key interactions resulted in accepting these differences in order to fit in.

It’s a new environment so the ability for you to adapt easily, and then have that teamwork with your team, because everybody is coming from different places, so with different attitudes. You are meeting people with different culture so there is this challenge of trying to learn other people’s culture, even if it’s not the same way you have known your culture; you have to compromise in order to go along (Peter).

**FIGURE 4:**
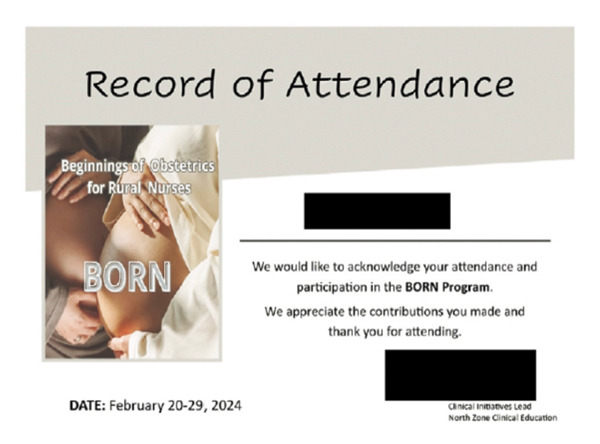
Participant’s title: “diverse, confident and versatile.” Photograph used with written consent.

The wider scope of practice also meant that IENs engaged in a significant amount of learning both independently by watching videos and reading online materials as well as participating in professional development opportunities sponsored by their employer. This type of support was highly valued by the participants and a clear vehicle to making their integration successful (Figure 4).

Education trainings in areas that I feel that I need more education I reach out to the education facilitator and then I engage in those education days. I have a lot lined up moving forward. And I will do more when there is more certification (Wendy).

*“Allowing themselves to be new”* (Wendy) by asking questions and receiving relevant responses and information created meaningful interactions for the participants. Both formal and informal mentorship, therefore, became an important vehicle for key interactions that had the potential to enhance their confidence in being able to provide safe, effective nursing care.

I worked with a guy, and I explained to him the first day that working back from home, I was working in a very big hospital and worked on a specific unit. Yes, I’m a registered nurse but I wasn’t in all the different departments in the hospital so there might be some things I might not know. And he said, “There is no problem.” He said, “I am willing to help you, if you are willing to learn.” I said, “Learning is not a problem, we keep learning every day.” Then after each day, we used to have our review time maybe 15, 30 min or 1 h and he would always have the time to sit with me and discuss and have a review of the day. So he really, really, really helped me (Possible).

There are some fantastic nurses who will say, “[Wendy], you’re good. I didn’t know you’re good like this. You’re good” (Wendy).

Moreover, having their previous knowledge and skills recognized and appreciated was especially important to the participants, which set them up for a successful transition and thus was a key interaction.

There are some things that I don’t know anything about, and that’s OK; I’m willing to ask questions and learn. But there are some things I do know about and for them to be able to recognize and say, “OK, maybe I can go and ask David about this” helps me to feel like I’m still contributing in that way (David).

That said, when more identifiable structures like a structured mentorship program or providing financial information aimed at supporting their integration into the workplace was missing or limited, the participants felt less supported.

If AHS would develop a structured preceptor program for IEN, they should structure it with their educational facilitators on how to integrate them into the system it should be structured so that it will fit into AHS standard. So that IEN comes in that you are given a booklet on what you are expected to learn today. Even if it is for two weeks, and then you are shadowed for those two weeks. Not based on your FTE or based on your orientation shadow that for this one week, this is how we are going to shadow you, and this is your learning pathway. Thank you for your knowledge, thank you for your experience, learn our own way of doing this, integrate it with your own so that you will give you better care. That’s what I want to see (Wendy).

And then finances are something that AHS didn’t quite prepare us for, which I don’t know if that was necessarily their responsibility, but it would have been helpful. (David)

**FIGURE 5:**
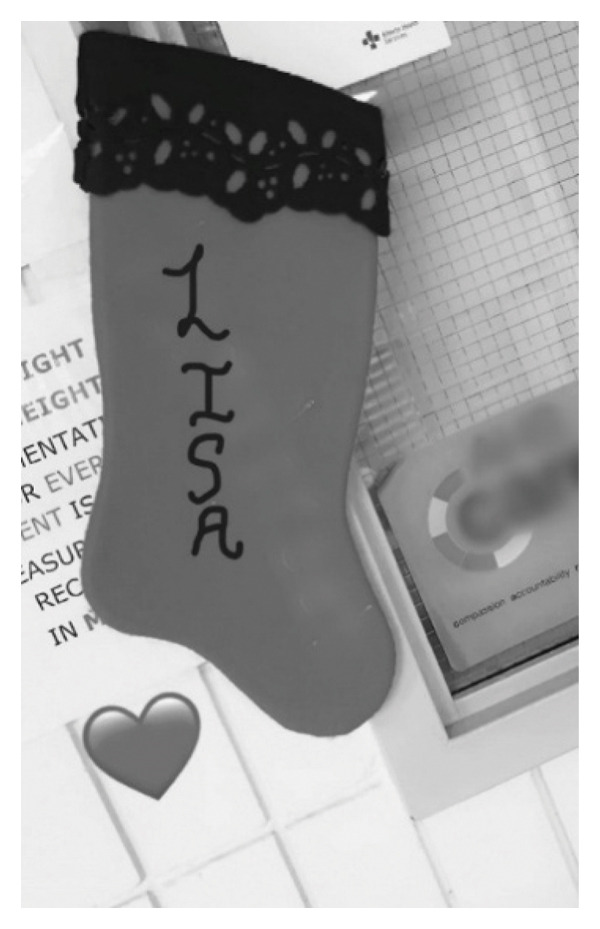
Participant’s title: “a special moment when I knew I belonged.” Photograph, with pseudonym, used with written consent.

It is important to note that verbal interactions were not the only way IENs experienced being supported. Nonverbal interactions were also very impactful especially when the message being sent by the domestic nurses was acceptance of the IEN (Figure 5).

That was the first time when I felt like they recognize us, they count us as a staff. That was the first time when I saw they had my name on there [referring to a Christmas stocking]. A few days back I was like “Will my name be there as well or just their names?” And there is my name as well. And that was like kind of they count us as well. That was the biggest happiness that I felt. But yes, they include us in their culture” (Lisa).

To extend how they were being supported, being able to connect with other immigrant nurses reinforced for the participants that they were not alone and that others understood what they were experiencing. This type of support points to the need for having a wide variety of supports from a wide variety of people even in the workplace (Figure 6).

Everybody I have asked questions to have answered my questions. But these two are kind of unique, they are willing to explain and still ask you – when you explain something to someone and that person is asking, “Do you really understand it now?” When you have somebody that has worked in this specific space before, that person has an idea, has the experience of whatever it is you are going through. The Canadians are nice, but they don’t have, they might not be able to relate with my present space, with what I am going through because they have never been in that situation. They have never been in that kind of space before. So that is why I have a bond, I’m more connected to people that have the experience, people that have worked in that space before. When you have people that have passed through that same line before, you learn to relate more. If you tell them one thing, they can think three things; they can figure it out quickly, to know, “OK, this is what she is trying to say. This is what she is trying to explain” rather than somebody that doesn’t even have any experience, doesn’t even understand what you are trying to pass across. So they give extra time to really explain things in‐depth. And they will say, “Oh, don’t worry, I know that things might be challenging now, but don’t worry, I was once like you.” And stuff like that. They were willing to share their experience. One of them came from, I think Jamaica, and the other came from the U.S. So it wasn’t easy for them when they first came, and they were able to relate with whatever it is I’m going through, or to foresee things that might happen in some weeks or some months time and to quickly push me through and prepare me on how to overcome some challenges. They have been able to fill the space, to fill the gap. If my mentor is not around, these are the two other people I’m comfortable speaking with and explaining and they will take their time to explain things and push me through. So, I can say they are my work buddies (Possible).

FIGURE 6(a) Participant’s title: “work buddies.” (b) Participant’s title: “a special bond.” Photographs used with written consent.

**Figure:**
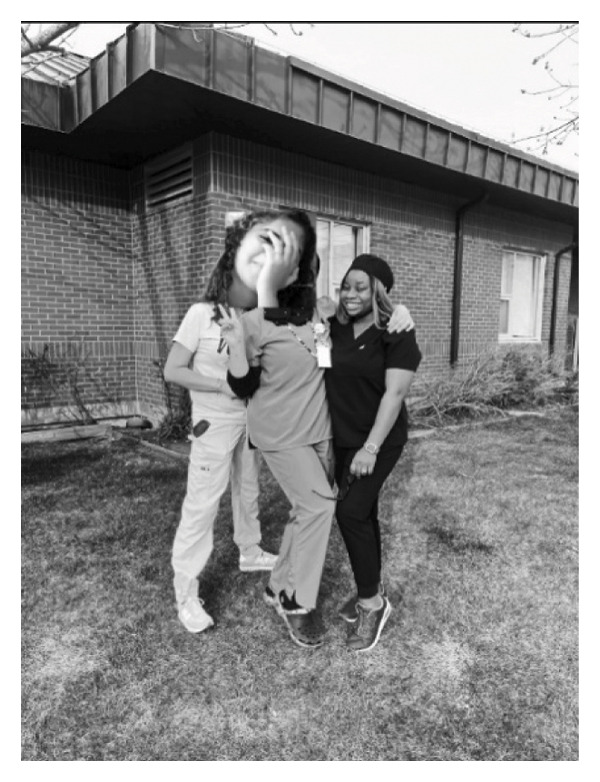
(a)

**Figure:**
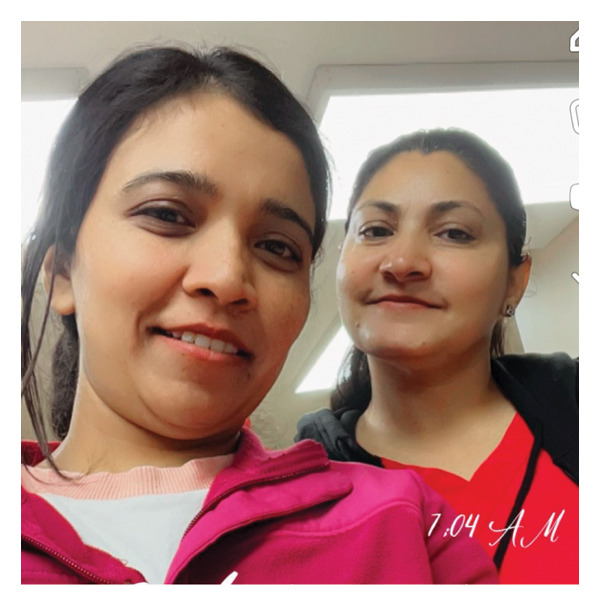
(b)

While most participants indicated they had positive interactions with members of the health care team, unfortunately, some participants felt socially and professionally isolated, and discriminated against. Lack of support leads to a deep sense of dissatisfaction and led to at least one IEN leaving her job and the rural community.

There are some people who don’t want to talk to you and do not feel like talking to you. There were some instances when people make you feel like you don’t belong. So at those moments it’s like kind of hard to communicate with a person, I felt somewhat like there was kind of like a gap between us, because I felt like, sometimes they were kind of trying to get away from [me], not really want to talk much. And that was – mentally, I did not feel good (Lisa).

When you go to the work environment it’s like you came to take people away from their position, and it’s like you came to grab, and there was no welcoming from the people you are to work with. For me as a person, it puts me off. It was like, “Did I make the right decision? Should I go back?” I see they were not prepared to have us. I’m not talking of the management, no. I’m talking about one‐on‐one interaction with your colleagues that you work with, as if they were not ready. As if they were not prepared for such a colour and, unfortunately for me, [they] haven’t seen more of variation (Wendy).

It is important to note that receiving support meant that interactions needed to be bidirectional. So, while the participants had expectations of their employer and new colleagues in welcoming them and how they would interact with them, they were also aware that they had a responsibility in developing interpersonal relationships with their colleagues even if they felt they were taking a risk.

I have also my own responsibility to try to develop interpersonal relationships with my colleagues. So I know that I have a lot to do, you need to get this because I need to get them to accept me. I try as much as possible to make sure I try to develop work relationship with as many as I work with per day, so that the environment is cordial and we will be able to work as a team (Wendy).

I know that I need to talk to people, and I need to know my limit and extent of whatever communication or conversation I’m having with people. I made it known to myself that I should open up to the extent that I know [it] will not come back to backfire on me. So that strategy of meeting with people, discussing challenges with people (Possible).

#### 3.2.2. Community Setting Interactions

Interactions with community members, especially those identified by AHS as key community members, that is, people with extensive knowledge and connections within the community (for example, church and religious leaders, business owners, law enforcement personnel, school principals, etc.), proved to be crucial in the participants’ transition to living in a rural community. Unexpected kind gestures from people they did not know were particularly appreciated by the participants.

The person at AHS gave me a contact person in the community. They said, “he can help you if you need to buy groceries, if you need to do this, he can explain stuff to you.” So, it actually made things easy for me. Whatever it is I need I have a key contact to speak to. It was the one way that introduced me to other people. I will say the guidelines AHS provided for newcomers, and then meeting people in the community are the two major things that helped me settle down fast (Possible).

I see heap of gifts dumped by my doorstep, things they knew I needed. I was like, “I never told you I need it, how come you know?” And then it’s there. Such little act of kindness and gestures was welcoming to us. We really, really appreciated it (Wendy).

So, while there was a bit of trepidation with moving to an unknown community, many participants spoke about “*knowing their neighbors*” which provided them with a sense of safety and sense of belonging.

The place is safe. You know your neighbours. You get to meet people and know them one‐on‐one. People can feel free and feel at home with you more than in cities where everybody’s like a foreigner. Everyone is afraid of his colleague or his or his community members because you’re like, “I don’t know him well”. It just takes few days or few weeks for you to start becoming acclimatized to the environment and then with the people (Peter).

The people are very kind. The people are very open. People have taken an interest in me and my family, interest in where we’re from, what we’re doing, why we’re here; these sorts of things. So all the interactions with people have been very positive. We’ve been able to create friendships. We’ve had people over to our house for dinner, and vice versa (David).

**FIGURE 7:**
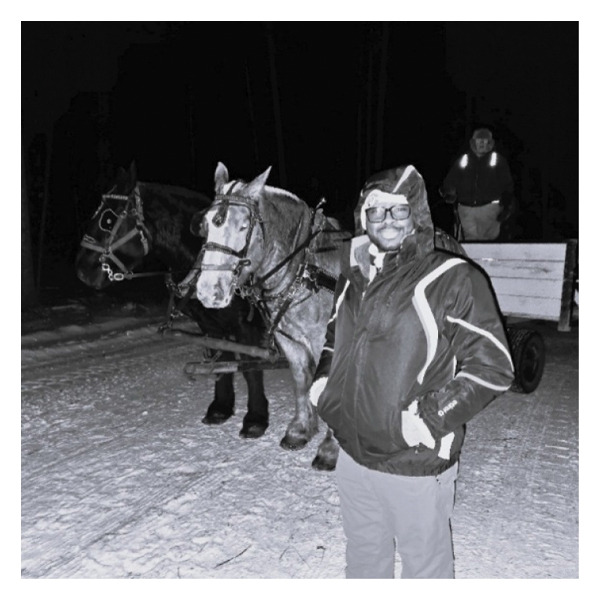
Participant’s title: “making community connections.” Photograph used with written consent.

Although community members seemed to be very receptive and supportive of the IENs, the participants also understood the need for bidirectional interactions which meant they needed to initiate interactions with the community members so that a relationship could be established. This sometimes meant becoming involved in sports activities, festivals, and volunteering with local organizations (Figure 7).

One of the things they [referring to recruitment officers] encourage us to do is be part of your community. Get engaged in the things they do. Get involved. So, they encourage us, “Don’t lock yourself inside, go out, interact.” And we do, as much as [possible]. We were able to transition into the community. I can see that happening (Wendy).

I felt that the best way to relate to people is to start knowing them. You can understand them better is to join them and see how they do their things, to associate with them when they are having their cultural anniversaries, just to be able to integrate properly and see how things are going with them. So it was very important to me because I think it helped me to associate with them, see how their culture is, the type of things they do, what they like, how they dance and also, it’s also – I see it as a way of fun, just having that fun, being around people, and enjoying the moment (Peter).

Still for others, connecting with other immigrant families in the community reinforced for them a sense of familiarity and another indication that support comes in a variety of ways. The participants also recognized that being rooted in family helped them make the transition to rural living.

I did to meet with the person that will be taking care of the kids. I did that with the parents of the other three children. And now we are like friends, because we share that in common. All of us are new. Although they have been here before me, they are not Canadians. So, we shared that in common. And I think I’m OK with their kids, the way they are training their kids as well. So, I’m comfortable that way (Possible).

Family gives you a sense of belonging. They have been my support base, and I don’t think I would have been able to hit it up running without them. The first day I went to work I lost my way. I was standing in the snow crying. I had to call my husband, I say, “I have lost my way, can you come and help me?” He took me in the hand and took me to work. Sometimes we need that family (Wendy).

**FIGURE 8:**
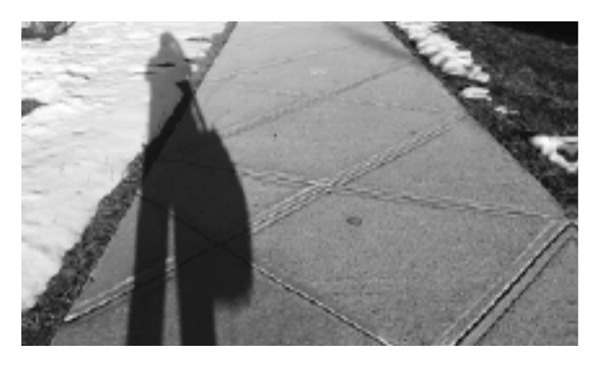
Participant title: “my shadow and me.” Photograph used with written consent.

However, making connections and engaging in interactions with community members was not easy for every participant. Consequently, being able to find another person who was experiencing similar challenges in integrating into the community was invaluable (Figure 8).

Honestly, it’s really hard to find friends in such a small community. Luckily, I met my friend, and we moved together, she got a position in [name of community] as well and we moved together. It was kind of – sharing with your day with someone is kind of a good experience. We spend lots of memories together, when we were getting emotional missing our family, we shared a bond (Lisa).

**FIGURE 9:**
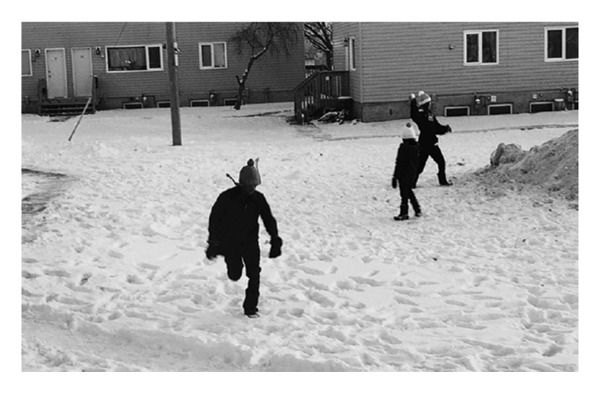
Participant’s title: “it takes courage.” Photograph used with written consent.

Lastly, some participants had an exit strategy if they felt they or their family was unwelcomed and or did not feel supported (Figure 9).

I promised, I said, “If you are not comfortable with and you decide you don’t want to stay, then you can come back home, if you want to.” I give him [referring to her son] the option that it’s not a matter of life and death. If at any point they feel that it is too much on them, then they can go back with their dad, and then I’ll finish up my tenure contract I signed and I will join them if it is too difficult for them. So, I gave them that option. I didn’t force it on them. I said, “This is another part of the world; you can still live here and enjoy it. Just understand them and understand their perspective and then you will be good. So, we are still working on it” (Wendy).

[In a] small town [it] is quite a task to find a job. He did try to move with me to [name of community] but because he does not speak much English, his English is not very good, he could not find anything, and he does not want to do something because of the language barrier. So he could not move with me, so we stayed apart (Lisa).

In summary, interactions at all levels of the healthcare system and with community members have the potential to influence IENs’ transition to and integration into rural nursing practice and living in a rural setting. Indeed, support that can be described as holistic, that is, comprehensive, extensive, and that meets each IENs’ unique needs is needed. If the interactions are inadequate and falls short of their expectations, leaving the workplace and community is a very real possibility.

## 4. Discussion

This study investigates how IENs encountered transitioning to and integrating into rural nursing practice and into their rural community. We found IENs needed to make important adjustments to feel comfortable and to “fit into” their new professional practice, workplace, and community. Thus, their transition and integration were anchored by critical interactions that created a sense of wraparound support, that is, support that was holistic and individualized bringing together community and professional resources. Their transition and integration were a dynamic process that included tactics, strategies, behaviors, and actions that led to a good fit between the IEN and their host environments. When the interactions and wraparound supports appeared to be missing or insufficient, the IEN became disillusioned, questioned their decision to immigrate and, in some instances, left their employment and community.

### 4.1. Workplace and Community Interactions and Wraparound Supports

IENs are highly motivated to become an “accepted and contributing member” of the nursing team and rural community. They are also highly committed to assisting their family members with their own transition and integration. To that end, IENs’ transition is highly dependent upon crucial interactions and connections with healthcare leaders and managers, nursing team members, and community members. However, as noted by Wellard and Stockhausen [30], there is a need for studies to explore the complex and varied issues and factors IENs encounter when transitioning and integrating into rural nursing practice, including barriers and challenges within clinical settings and community environments. The broader IEN literature also echoes the need to advance our understanding of cross‐cultural challenges and cultural differences [31], and social integration and establishment of connections [32].

The findings in this study highlighted that IENs relied on a variety of workplace and community interactions during their transition to adjust to the multidimensional cultural differences and to “fit in.” Bidirectional, reoccurring interactions with healthcare professionals and community members that included compromise, respect, acceptance, and professional practice knowledge translation and dissemination provided the foundation for successful integration. By leveraging their networks (e.g., managers, nurse colleagues, and work buddies), IENs appeared to develop an understanding of the cross‐cultural and professional differences, values, and practices as well as intercultural communication mechanisms in the workplace. Although they also had to adjust to a wider scope of practice and increase responsibility, to become part of the nursing team, they needed to understand and adjust to the differences in rural nursing practice compared to what their nursing practice in their home country was like. Consequently, they used interactions, tactics, and strategies such as asking questions, shadowing nurse colleagues, seeking mentorship, engaging in formal and informal professional development, and developing a buddy relationship with other IENs to gain the necessary knowledge and skill to engage in the full scope of practice of rural RNs. Conversely, the IENs engaged in cross‐cultural dissemination by sharing their home country’s nursing standards and practice requirements with their managers and nursing colleagues thereby demonstrating a strategy to build cross‐cultural awareness of different models of nursing practice. The interactions IENs had with their nursing team members created a sense of support and enhanced their professional confidence to adjust to the demands of rural nursing.

IENs spoke of their deliberate, strategic approach they used to “fit” into the rural community. They approached their transition to “fit in” with a plan. IENs understood they needed to leverage their interactions with community members by getting involved in community activities. IENs interacted and connected with community members while watching their children participating in sports, during church services, and volunteering and asking for contact names. Another strategy was to develop an understanding of community members’ cultural perspectives, activities, holidays, and practices. They were also very willing to accept whatever support community members offered. As noted by Foo [31], the active involvement of community members in IENs’ social integration is an important component. However, it is important to note that even though their work integration might have been unfolding satisfactorily, IENs like those in this study might have an exit plan in the event they feel, or their family members feel unwelcomed and do not belong. These findings extend the literature by highlighting IENs’ social integration is a complex process dependent upon IENs’ leveraging social capital by building relationships, and by bridging and understanding cultural differences and similarities.

It was evident to us that wraparound supports included both formal activities/actions (e.g., recruitment materials, assigned mentors, clinical educators, orientation program, professional development, and introduction to community member) and informal supports via their interactions with other IENs, nursing colleagues, patients, faith leaders, and new acquaintances including other immigrants. Both types of wraparound supports are essential and appear to have a positive impact on IENs and their family’s transition and integration by creating a sense of becoming part of the nursing team and belonging in the community. When formal wraparound supports were missing in the workplace, IENs expressed the need for a formalized orientation and onboarding (e.g., understanding AHS practice standards, required nursing competencies, and preceptorship) and a formal, detailed transition plan for nursing practice (e.g., mentorship, shadowing, individualized learning outcomes, and professional development opportunities). Additionally, when wraparound supports in the community, such as information about cost‐of‐living, financial matters, transportation, primary educational system, housing, and employment opportunities for spouse were limited or missing their transition and integration were much more difficult.

### 4.2. Recommendations for Supporting IENs’ Transition and Integration

Healthcare leaders and unit managers who demonstrate positive leadership are pivotal in developing cross‐cultural, inclusive workplaces and reducing the cultural and professional distance between IENs and their nursing colleagues both in the short term and the long term. The extent literature reports various organizational factors, such as wraparound supports, targeted training programs, and mentorship, that enhance IENs’ sociocultural adaptation [33]. Additionally, organizational policies, culturally tailored onboarding, and ongoing professional development (e.g., language barriers, unfamiliar social norms, differences in healthcare practices, iterative feedback, engagement of community members, and cultural mediators/interpreters) also support IENs’ adaptation [34] and should lead to reducing misunderstandings, aligning expectations, and reducing resistance to unfamiliar norms for all. Ultimately, healthcare organizations need to create open‐mindedness towards developing and maintaining cultural‐sensitive work environments.

#### 4.2.1. Key Recommendations for the Transition and Integration of IENs Into the Workplace and Rural Community in Alberta

There are several key recommendations that flow from the findings of this study and that inform the recruitment and potential retention of IENs who choose to work in rural settings. These recommendations are consistent with adopting a wraparound support approach. That said, each rural community and practice site is unique. These recommendations are intentionally broad and need to be adjusted to meet the unique needs of individual communities and rural workplaces.

• We involve the rural healthcare sites in the recruitment of IENs with the intent to identify “fit” and to initiate the development of interactions and connections.
• We prepare the rural healthcare sites for the arrival of IENs by involving the sites in the development and implementation of formal orientation and mentorship or preceptorship programs, welcome activities, and introduction sessions for hospital staff and community members.
• We provide IENs and host staff members training in unconscious bias and cultural sensitivity topics plus training in the role of professional and social interactions and connections.
• We develop a competence form that outlines practices skills and knowledge unique to rural nursing for the assessment of IENs’ competences against professional standards.
• We prepare a unique learning plan for each IEN with the aim to address professional knowledge and skill gaps.
• We prepare education materials and offer a variety of professional development opportunities tailored specifically to the needs of the IENs.
• We develop an IEN buddy program and build a community of IENs as a support network.
• We provide a comprehensive information package detailing cost of living, housing, childcare, public education, spousal employment opportunities, transportation, churches, and amenities unique to each rural community.
• We introduce and connect IENs with key members in the rural community.

### 4.3. Limitations, Strengths, and Future Research

A limitation of this study is the sample consisted of seven IENs’ experiences as they transitioned into rural nursing practice in care facilities in Alberta, Canada. Although it is not unusual to have a smaller number of participants when conducting rural research, the small sample size is likely a reflection of the constraints put on the recruitment processes. For example, the recruitment process was limited to the Chief Nursing Office disseminating study invitations to IENs. Moreover, invitations to participate in the study were limited to IENs working in only 15 out of 75 possible AHS rural sites. To date, there is no provincial process for obtaining organizational approval for province‐wide research projects. A second limitation is these rural sites were in two geographical regions, central and/or northern Alberta, which might impact the generalizability and applicability of the findings to other rural locations and geographical areas. For example, IENs’ rural experiences in southern Alberta, with its higher population density and closer proximity to urban centers, might be different than IENs’ experiences in northern rural communities. Two participants had rural experience in their home countries; therefore, their experience might have influenced their experiences in rural nursing practice in Alberta. Lastly, because, to date, only RNs are being recruited by AHS, the interview sample was limited to internationally educated RNs. Including internationally educated licenced practical nurses’ perspectives and experiences would help to deepen our understanding of working in rural settings.

This study has three primary strengths: (1) its rich, deep, and nuanced qualitative dataset; (2) findings that are consistent with results in the published literature; and (3) other findings that provide new insights about IENs’ transition and integration adjustments (e.g., tactics, strategies, behaviors, and actions) and sociocultural adaptations (e.g., behavioral skills and acculturation strategies). This study starts to address significant gaps in the research about the crucial, reciprocal roles of IENs who are taking responsibility for developing their interpersonal connections and relationships with their colleagues, managers, and community members; conversely, those individuals play an essential role in IENs’ transition and integration by demonstrating acceptance, collegiality, and support.The findings from this study most likely will generalize to other rural and remote areas within and outside of Canada. Finally, some of the results from this study add to literature by highlighting the multidimensional nature of tactics, strategies, behaviors, and actions. IENs utilize and how a wide variety of supports infuse their experiences of becoming a valuable and effective member of the nursing team and rural community.

Future quantitative designs or mixed methods studies could explore the potential associations among IENs’ interactions with professional colleagues and community members, the impact of wraparound supports, and their intention to stay in their rural nursing position or leave for another nursing position in an urban area. A longitudinal study could capture potential changes in the experiences of IENs over time. Additionally, a comparative study could offer valuable insights into how IENs experiences in rural settings differ from their experiences in urban or other rural settings. Exploring organizational leaders’ and healthcare team managers’ interactions with IENs during their transition and integration would add to the body of literature. Lastly, since families play an integral role in IENs’ transition and integration into the rural setting, a study that explores family members’ experiences would help deepen our understanding of this experience.

## 5. Conclusions

The Canadian nursing workforce continues to experience a critical shortage of nurses, especially in rural settings where significant threats to the sustainability of healthcare services are evident. The findings from this study suggest that IENs recruited to work in rural settings experience and engage in critical workplace and community interactions, and they use a variety of professional and sociocultural tactics, strategies, and actions during their transition and integration. They are highly motivated to “fit into” their rural nursing practice and community setting and to be accepted as valued, contributing members of the nursing teams and community. Additionally, IENs’ transition and integration are contingent upon the pivotal roles of healthcare leaders, managers, nursing colleagues, and key community members. These interactions create wraparound supports leading to the creation of welcoming environments that reduce the cultural and rural practice distance between IENs and their nursing colleagues and community members.

## Funding

This research project was funded by Rural Health Professions Action Plan (RhPAP).

## Ethics Statement

This research was approved by the University of Alberta Research Ethics Office (Pro00137602) and received operational approval from AHS.

## Conflicts of Interest

The authors declare no conflicts of interest.

## Endnotes

^1^The largest provider of healthcare services in Alberta, Canada.

^2^The professional licencing body for regulated nurses in Alberta, Canada.

## Supporting Information

Participant Consent Form‐Interview.

## Supporting information


**Supporting Information** Additional supporting information can be found online in the Supporting Information section.

## Data Availability

The data that support the findings of this study are not publicly available due to privacy or ethical restrictions.
