# Supplementary material for: Wraparound Supports: The Key to the Transition and Integration of Internationally Educated Registered Nurses in Rural Alberta, Canada
Source: J Nurs Manag. 2026 Apr 21;2026:7378466. doi: 10.1155/jonm/7378466 (PMC13096793; doi:10.1155/jonm/7378466)
Supplement: Supplementary file 1 — Supporting Information Additional supporting information can be found online in the Supporting Information section. [file JONM-2026-7378466-s001.docx]

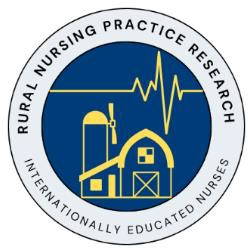

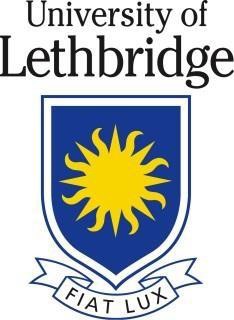
4401 University Drive Website: [www.ulethbridge.ca](http://www.ulethbridge.ca/) Lethbridge, Alberta, Canada Phone: (403) 329-2111

T1K 3M4

# PARTICIPANT CONSENT FORM - Interview

**Title of Study:** Internationally Educated Nurses’ Experience of Rural Alberta Nursing Practice: A Mixed Method Study

**Principal Investigator:** Dr. Monique Sedgwick, Associate Professor, Faculty of Health Sciences, University of Lethbridge. Email: [monique.sedgwick@uleth.ca;](mailto:monique.sedgwick@uleth.ca) Phone: 403-329-2432

**Co-Investigators:** Dr. Helen Kelley, Associate Professor, Dhillon School of Business, University of Lethbridge Dr. Daniela Sirbu, Associate Professor, Faculty of Fine Arts, University of Lethbridge

**Project Manager:** Ms. Michaela Schultchen, MA, Program Specialist – Curriculum & Timetabling, Faculty of Health Sciences, University of Lethbridge.

**Why am I being asked to take part in this research study?** You are invited to participate in this research study about Internationally Educated Nurses’ (IENs) experiences in rural practice settings, and strategies you use to support your resilience and ability to thrive in your rural practice environment. Your participation provides an opportunity to express via photographs and to describe your rural experiences working in a rural practice setting.

**What is the purpose of this form?** This form contains information about the study. Before you read it, a member of the study team will review the detailed information explaining the study that was shared during the research orientation session. You are free to ask questions about anything you do not understand and/or want additional information about. You will be given a copy of this form for your records.

**What is the reason for doing the study?** From this research, we wish to understand IENs’ experiences as well as their adjustment to and integration into the healthcare system when working and living in rural areas. We will

explore the strategies IENs use to support their resilience and ability to thrive in their rural practice environment.

Additionally, we want to learn how IENs’ experiences, adjustment, and integration influence their career aspirations, intent to stay, family/work life balance, and sense of community. The research will also include

several evaluation questions asking about Alberta Health Services’ (AHS) pilot program for rural Alberta nursing practices.

This is a multi-phased study involving several rural settings across the North, Central and South Zones of AHS. Members of the research team will interview 18- 20 IENs participating in the photovoice component of this

project. Following the analysis of the photos, interviews, and focus group, guides/mentors, managers, nurses, and other healthcare professionals who interact with IENs will be invited to complete a survey asking for their

assessments of AHS’ pilot program. Other IENs will also be invited to complete a survey.

**What will I be asked to do?** You will take 5- 20 photographs (or as many as you wish) using your mobile phone of events, things, and/or interactions with colleagues and patients that represent what practicing nursing in rural Alberta means to you. Once you have taken all the photographs you feel are important, you will select five (5) photographs that best represent your experience of rural nursing practice and email them along with some brief notes, to the research team using the following email address [international.nurses@uleth.ca](mailto:international.nurses@uleth.ca)

Once your pictures and brief notes have been received, a member of the research team will contact you to schedule an interview using Zoom at a time that is mutually convenient. During the individual interview, using the five (5) photos you sent to the team, you will be asked a set of open-ended questions such as:

1. The meaning and relevance of each photograph (e.g., what do you see; how does this situation relate to your experiences in rural nursing practice, etc.);
2. The importance of each photograph to your experiences and/or challenges in a rural practice environment;
3. Your experience of working and living in a rural community and;
4. Demographic questions

Each interview will last approximately 60 minutes depending on your responses. You will be interviewed only once. The interviewer will record your interview with your consent and will take notes during the interview. The audio recording of your interview and interview notes will be transcribed for analysis purposes. The audio recording, notes and the interview transcription will be stored in a password-protected folder of the research

team’s Dropbox account that only members of the research team and the transcriber can access.

You will also be invited to participate in one of three focus group interviews with other IENs who have taken photographs and completed the individual interview (there will be between 6-8 other participants in the focus group interview). The focus group interview will be virtual and recorded. We anticipate the focus group interview to take about 60-to-120 minutes.

**How long will I be in this study?** Your participation in the study will involve about 13.5 to 15.5 hours of your time over a six-month window to take photographs and to participate in the interview, focus group, and online survey.

**What are the risks and discomforts?** We do not anticipate any known risks associated with this research. It is not possible to know all of the risks that may happen in a study, but we have taken all reasonable safeguards to minimize any known risks to a study participant. No identifiable information will be shared with AHS or any other employer.

**What are the benefits to me?** You will be helping to advance scientific knowledge about IENs’ experiences and challenges working and living in rural areas and with their rural clinical practice. Additionally, we will develop an understanding of the strategies IENs use to support their resilience and ability to thrive in their rural practice environment. What we learn will help to guide work towards improving IENs’ integration into rural healthcare

settings; inform policy and practice processes and protocols that are culturally sensitive, supportive, and relevant for IENs; advance recruitment and training and; enhance rural clinical practices and outcomes of rural Albertans’ healthcare.

**Do I have to take part in the study?** Being in this study is your choice. You do not need to answer any question you do not feel comfortable answering. If you decide to be in the study, you can change your mind and stop being in the study by contacting Dr. Sedgwick within five days of your individual interview. If you decide to withdraw from the study, the audio recording of your individual interview will be destroyed. If you start but decide to leave the focus group, you simply need to leave the meeting. Data shared to that point will not be able to be removed from the audio recording of the focus group.

**Will I be paid to be in the research?** You will receive a $60.00 Figs gift card (i.e., scrubs) for participating in the qualitative component of this study – photographs, notes, interview, and focus group. If you decide to withdraw from the study before the end of the interview, you will still receive the $60.00 gift card.

**Will my information be kept private?** During the study we will be collecting data about you. We will do everything we can to make sure that your data are kept private. No data relating to this study that includes your name will be released outside of the researcher’s office or published by the researchers. Sometimes, by law, we may have to release your information with your name so we cannot guarantee absolute privacy. However, we will make every legal effort to make sure that your information is kept private.

There will be other participants during the focus group who will see your face. You can turn your camera off if you wish but the other participants will still hear your voice and comments (you will have to turn your mute button off). At the start of the focus group interview, we will ask all the participants to keep the discussion confidential and when possible, to refrain from using names of specific people, communities, or hospitals when discussing their experiences. We will also remind participants that all information shared during the focus group interview is confidential and cannot be shared outside of the focus group interview.

Your individual interview and the focus group interview will be audio recorded only and stored directly onto a password-protected folder of the research team’s Dropbox account that only members of the research team and transcriptionist can access. The recording will not be stored on the Zoom cloud. Your name and any other identifiers will not be included in the interview and focus group transcripts.

**What if I have questions?** If you have any questions about the research now or later, please contact Dr. Sedgwick via email at [monique.sedgwick@uleth.ca.](mailto:monique.sedgwick@uleth.ca)

If you have any questions regarding your rights as a research participant, you may contact the University of Alberta Research Ethics Office at [reoffice@ualberta.ca.](mailto:reoffice@ualberta.ca) This office has no affiliation with the study investigators.

None of the members of the research team have potential conflicts of interests with the funding provider for conducting this study or with being involved with any part of this study and/or the possibility of commercialization of the findings.

## How do I indicate my agreement to be in this study?

By signing below, you understand:

- That you have read the above information and have had anything that you do not understand explained to you to your satisfaction;
- That you will be taking part in a research study;
- That you may freely leave the research study by the specified time;
- That you do not waive your legal rights by being in the study; and
- That the legal and professional obligations of the investigators and involved institution are not changed by your taking part in this study.

## CONSENT TO PARTICIPATE IN THE FOLLOWING RESEARCH PROCEDURES

Initial all that apply:

: Taking photographs

: Having photographs displayed in the public domain (presentations, articles, exhibits, etc)

: Participating in an individual interview

: Participating in a focus group interview

## SIGNATURE OF STUDY PARTICIPANT

By checking this box and inserting my name below, I am electronically signing this consent form

Name of Participant Date

## SIGNATURE OF PERSON OBTAINING CONSENT

Name of Person Obtaining Consent Contact Number

Signature Date

A copy of this consent form will be sent to you to keep for your records and reference.


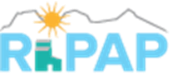


Funded by Rural Health Professions Action Plan (RhPAP)
